# Supplementary figures and images for: Acetylation of the yeast Hsp40 chaperone protein Ydj1 fine-tunes proteostasis and translational fidelity
Source: PLoS Genet. 2024 Dec 9;20(12):e1011338. doi: 10.1371/journal.pgen.1011338 (PMC11658694; doi:10.1371/journal.pgen.1011338)

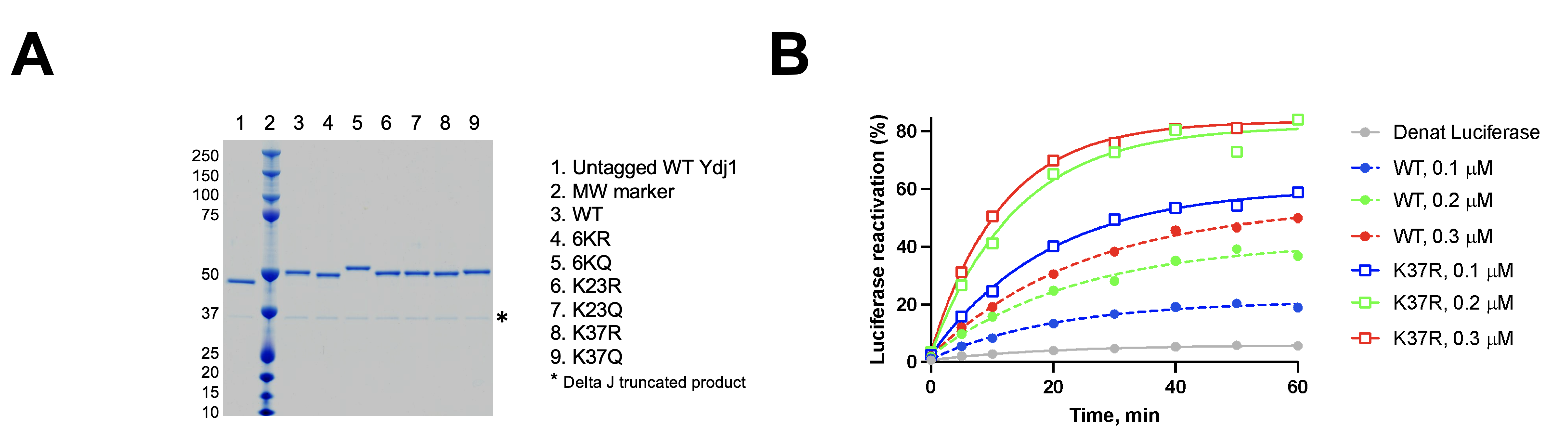

Supplement: S1 Fig — (A) SDS PAGE of purified Ydj1 wild-type and mutants. 0.5 μg of purified His-tagged Ydj1 wild-type and mutant proteins were run on a 4–12% NuPAGE gel (Invitrogen, Thermo Fisher Scientific) using MOPS running buffer. 0.5 μg of purified untagged Ydj1 wild-type was included for comparison. The gel was imaged following staining in InstantBlue Coomassie Protein Stain (Abcam). Molecular weight marker sizes (kDa) are indicated. (B) Reactivation of chemically denatured luciferase by Ssa1 and Ydj1 wild-type or K37R using a second preparation of His-tagged Ydj1 wild-type or mutant. Assays were performed as in Fig 6A. Results are shown as means ± SD of three replicates. (TIFF) [file pgen.1011338.s001.tiff]

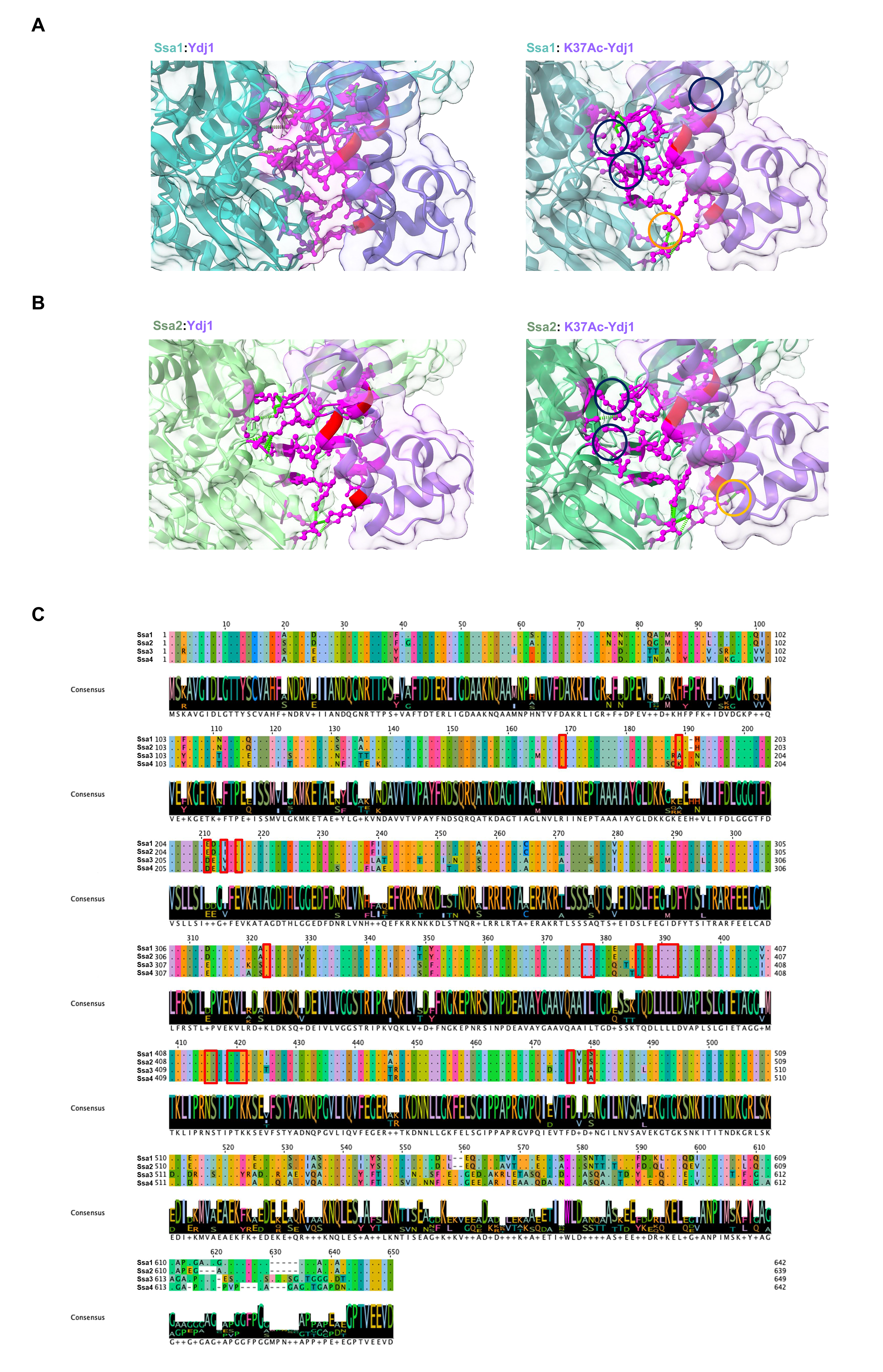

Supplement: S2 Fig — (A) Predicted interaction surface between Ssa1 and K37 acetylated Ssa1 with the Ydj1 J-domain. The interaction surface is highlighted (magenta), along with respective hydrogen bonding (lime green hash marks). Structures were obtained via the Alphafold 3 Server and mapped and characterized using ChimeraX. (B) Predicted interaction surface between Ssa2 and K37 acetylated Ssa2 with the Ydj1 J-domain. The interaction surface is highlighted (magenta), along with respective hydrogen bonding (lime green hash marks). Structures were obtained via the Alphafold 3 Server and mapped and characterized using ChimeraX. (A) Ssa1-4 Amino acid alignment and consensus graph as determined by Jalview, the red boxes indicate sites of interaction on Ssa1 with WT Ydj1 modeled with AlphaFold 3. (TIF) [file pgen.1011338.s002.tif]
