# Supplementary material for: Acetylation of the yeast Hsp40 chaperone protein Ydj1 fine-tunes proteostasis and translational fidelity
Source: PLoS Genet. 2024 Dec 9;20(12):e1011338. doi: 10.1371/journal.pgen.1011338 (PMC11658694; doi:10.1371/journal.pgen.1011338)
Supplement: Attachment — Submitted filename: Response to reviewers PLOS GENETICS Ydj1 9a oct.pdf [file pgen.1011338.s006.pdf]

UNIVERSITY OF NORTH CAROLINA
CHARLOTTE
BIOLOGICAL SCIENCES

Department of Biological Sciences
9201 University City Boulevard, Woodward 486C
Charlotte, NC 28223-0001

Tel: (704) 687-5228
e-mail: atruman1@uncc.edu

Andrew W. Truman-PhD

October 8, 2024

Dear Editor,

We offer below our point-by-point responses to queries and comments on our submitted manuscript titled "Acetylation of the yeast Hsp40 chaperone protein Ydj1 fine-tunes proteostasis and translational fidelity". We would like to thank both you and the reviewers for your constructive comments and positive feedback. The senior author of this manuscript is currently on maternity leave and has come back early in a very limited capacity to address these reviews. Despite these challenges, we have substantially revised the text and added several new figures/data while also improving the quality of the existing ones. While we understand that further studies would reveal more of the underlying mechanisms controlling Ydj1, we believe these data satisfy the requirements for publication in *PLOS Genetics*.

Response to Reviewer 1 comments

"The Chaperone Code describes the complex post-translational regulation of molecular chaperone proteins that confers selectivity and specificity to chaperone function and chaperone-substrate interactions. High-throughput studies have identified numerous chaperone modifications, however functional information is lacking. Here, the authors have made significant progress in understanding lysine acetylation on the major Hsp40 chaperone Ydj1 in yeast. While the manuscript contains some strong and compelling data, additional work is required to support the major claims of the study".

We thank the reviewer for their positive comments.

"*Despite the framing of this paper around Ydj1 acetylation, the authors have not experimentally validated Ydj1 acetylation at any point in the manuscript. This is absolutely essential to support the interpretation of the results".*

We selected the six residues to study based on their previous detection in multiple proteomic studies. As explained in the text of the manuscript this can be viewed here: https://intrinsicdisorder.com/a/ptm_png.py?I=YNL064C. Each of the six acetylation sites have been detected between 17 and 42 times. We have studied chaperone PTMs for the better part of twenty years. Our typical methodology involves identifying interesting sites and then studying 1) their impact by mutation and 2) their regulation using PTM-specific antibodies. We initially attempted to analyze the six sites using general acetyl-lysine antibodies, but the data were confounded by the poor quality of the antibody and multiple acetylated sites. We then attempted to generate site-specific acetyl-lysine antibodies for Ydj1 twice at great cost and time ($5000 per antibody with a nine months generation time). Disappointingly none of these antibodies were selective enough for us to use, they simply detected total Ydj1. As such, we ended up with the analysis we present here. We believe that the identification of these sites by MS multiple times (in both yeast and human cells) is sufficient for this study.

"*The authors state in the results section that Rps0A levels are moderately reduced in cells expressing the 6KQ mutant. However, this is unclear from the presented data. It is necessary to replace the Rsp0A blot in Fig 5C with a lower exposure to appropriately evaluate the expression levels.*"

We agree with the review and have replaced the blot with a lower exposure. In addition, we have performed quantitation of the blots and have represented the ratio between RPS and loading control signal on the revised figure.

"*The authors have consistently used the 6KR/6KQ mutants throughout the manuscript, however much of the change in activity is derived from mutation of K23 or K37. Perhaps a double mutant of these two residues would be more representative of Ydj1 acetylation state, and mitigate the potential effects of mutations at the other 4 sites. A more thorough exploration of different mutation combinations is necessary*".

Our initial analysis began with the study of the phenotype of individual site mutants and then the combined 6KQ/6KR. These experiments overall suggested that K23 and K37 cause the majority of the effects and our *in vitro* experiments confirm this. While combinations of other mutants may be of interest in future studies, we don't think this would add to this particular story.

"*Similarly, it is unclear why the authors did not test binding of the 6KQ and 6KR mutants in Fig 6C. This would seem to be essential for correlation with the rest of the data in the manuscript*".

We thank the reviewer for their suggestion. The results with 6KR and 6KQ have been added to Fig. 6.

"*In the manuscript the authors have concluded that "the main phenotypic defects seen in the KQ mutants are likely a result of the loss of ability to bind Ssa1 and stimulate the Ssa1 ATPase activity." However, the authors have performed an interactome and identified many proteins with differential binding to Ydj1 acetylation mutants. Though the authors state that Ydj1 acts through Hsp70/Hsp90, have the authors considered direct effects of Ydj1 activity? In order to support this claim, have the authors determined whether the reported interactors identified in Fig 4B known to rely on Hsp70/Hsp90 chaperoning? If the authors would like to support this conjecture, it would be useful to IP Ssa1 from yeast expressing Ydj1 mutants and repeat the global proteomics, in addition to the interactome of Ydj1 itself. In fact, the observation that the 68% of Ydj1 interactions were not disrupted by acetylation mutants could suggest that these changes are actually independent of Ydj1 regulation of Ssa1*".

We thank the reviewer for bringing up this point. Ydj1 binds clients and transports them to Ssa1 for folding in addition to stimulating Ssa1 ATPase activity. For this revision, we analyzed how many of the Ydj1 interactors changed by prevention of acetylation are known interactors of the Hsp90/Hsp70 system. Out of the 120 acetylation-dependent Ydj1 interactions observed, only 21 are not known to be clients of the Hsp70/Hsp90 system. The Venn diagram showing this is now shown as part of Figure 4.

We have revised the discussion to consider this point.

"*Related to the previous point, in order to tease out the impact of Ydj1 acetylation on chaperone dynamics, the authors should coIP Hsc82/Ssa2-4 in Fig 4E. If no specific antibodies for these isoforms exist, the authors could also express the different Ssa isoforms as the sole copy of Ssa in yeast (as they have done previously (Omkar, 2022)) and repeat Fig 4E in those cells.*"

We agree with the reviewer that an Hsc82-Ydj1 co-IP would be of benefit to validate the MS data. We have completed this experiment, which validates the result of our proteomic study. This can now be found as Figure 4F.

"*P11, line 237 should be Figure 5D, not 6D*".

The text has been adjusted to reflect the change from 6D to 5D.

"*The authors should also mention the possibility of Lys PTMs aside from ubiquitination (e.g. SUMOylation) as potential modifiers of these conserved Ydj1 residues that would influence the chaperone interplay in higher eukaryotes*".

We thank the reviewers for bringing up this point especially as the interplay between adjacent PTMs and PTMs on the same amino acid are a topic of great interest to my group. We have altered the text to mention the possibility of multiple modifications.

Response to Reviewer 2 comments

The manuscript by Omkar S. et al. examines the role of lysine acetylation modification on the J-domain of Ydj1. The 12 acetylation sites were identified using the GPM database, and further work focused on the 6 sites present at the J- domain of the protein. The authors demonstrate that Ydj1 mutants that mimic non-acetylation support yeast growth like that of wild-type Ydj1 in response to a number of stress conditions. Among various mutants, the acetylation mimics of Lys23 and Lys37 poorly supported yeast growth against thermal stress. Further, the 6KQ mutant showed a loss of function phenotype against all stressors examined in the study. The authors further carried out an interactome study to examine the effect of acetylation on interaction with other cellular proteins, and found that acetylation affects Ydj1 interaction primarily with those involved in translation. The study further explored the role of Ydj1 acetylation in the stabilization of ribosomal subunits. Overall it's an important study that uncovers the role of Ydj1 acetylation on its native function and opens up various interesting possibilities to examine the role of PTMs on Ydj1 in the maintenance of protein homeostasis.

We thank the reviewer for their positive comments on the work.

"The primary focus of the manuscript is to examine the significance of Ydj1 acetylation in its in vivo roles. However, no attempts are made to show that the co-chaperone is acetylated under any of the stress conditions examined. The authors should provide evidence of acetylation under at least one of the stress conditions examined".

We selected the six residues to study based on their previous detection in proteomic studies. As explained in the text of the manuscript this can be viewed here: https://intrinsicdisorder.com/a/ptm_png.py?l=YNL064C. Each of the six acetylation sites have been detected between 17 and 42 times. An important point to note is that throughout our twenty-year study of the chaperone code we see clear evidence that sites that impact specific stress resistance are intriguingly often not those that are activated by the same stresses. This is possibly due to multi-pathway crosstalk and integration. However, our typical methodology involves identifying interesting sites and then studying 1) their impact by mutation and 2) their regulation using PTM-specific antibodies. We initially attempted to analyze the six sites using general acetyl-lysine antibodies, but the data were confounded by the poor quality of the antibody and multiple acetylated sites. We then attempted to generate site-specific acetyl-lysine antibodies for Ydj1 at great cost and time ($5000 for each one with a nine-month generation time). Disappointingly none of these antibodies were selective enough for us to use, they simply detected total Ydj1. As such, we ended up with the analysis we present here. We believe that the identification of these sites by MS multiple times (in both yeast and human cells) is sufficient for this study.

"Figure 4E: The 6KQ mutant exhibits a weaker binding affinity to Ssa Hsp70 than the wild-type Ydj1. The binding of 6KR is much stronger. It would be uncommon for all six lys to undergo acetylation

simultaneously in vivo. It is thus important to identify specific acetylation site(s) (among the 6 lysines) that affect Ydj1 interaction with Ssa".

While we certainly believe that K23 and K37 are the major regulators of Ydj1-Ssa1 interaction (as seen by the in vitro experiments), there is no reason to believe that all six could not be acetylated simultaneously. However, we have revised the text to address this point.

"Figure 5C: The lowered abundance of Rps0A, Rps3 and Gcd11 could be due to an effect on transcription. Authors should explore this possibility".
A major benefit of using the yeast ORF plasmid collection is that they are all controlled by the galactose promoter. We have used this collection many times throughout the years as the constitutive promoter prevents any confounding transcriptional issues.

"Page 11: "whereas the 6KQ and K37Q exhibited ~ 2 fold……...to wild type (Figure 6A)". The mentioned mutants 6KQ and K37Q are most likely 6KR and K37R (needs to be clarified). Additionally, what could be the reason for the increased activity of these mutants in comparison to the wild-type protein, given that the wild-type protein is also not acetylated?

We thank the reviewer for pointing out this error and have corrected it. The data show that 6KR and K37R are hyperactive in reactivation. A few sentences have been added, "The explanation for the increase in reactivation activity by the 6KR and K37R mutants is not known. The results were confirmed using a second preparation each of purified Ydj1 wild-type and K37R (Figure S1C). One possible explanation for the hyperreactivation activity is that the two mutants bind Ssa1 more weakly than wild-type (as is shown below in Fig. 6C), thereby allowing a faster rate of turnover and reactivation."

6KR has increased interaction with Ssa1 however K37R has similar interaction with Ssa1, and thus increased activity seems to be unrelated to changes in interaction with Ssa1".

The binding of Ydj1 WT, K23R and K23Q to Ssa1 are all within experimental error. We have added error bars to Fig. 6C. We mentioned in the response above that we do not fully understand why 6KR and K37R are hyperactive in luciferase reactivation. It is clear that in vitro K6Q and K37Q are unable to interact with Ssa1, unable to reactivate luciferase with Ssa1 and defective in stimulating Ssa1 ATP hydrolysis.

"*Page 12: Authors mention that stimulation of Ssa1 activity by 6KQ and wt Ydj1 is similar. However, Figure 6B shows that wt Ydj1 is significantly more active. This needs to be clarified.*

The 6KQ typo has been corrected to "6KR".

Response to Reviewer 3 comments

"In Figure 3 the argument is made that doublet protein bands visible by Western blot can be attributed to farnesylation. However, the C-terminal FLAG tag would cause interference with farnesylation. This does not change the main conclusions of the paper, but any comments about Ydj1 doublets with respect to farnesylation need to be significantly revised or removed. See Lines 140 and 366".

There have been quite a few studies on Ydj1 farnesylation over the years and fortunately C-terminal tags do not impact farnesylation (Flom et al. 2008; Sojourner et al. 2018; Caplan et al. 1992).

"Figure 5A and 5C and its interpretation: The authors observe that 3 proteins (Rsp0A, Rps3 and Gcd11) have lower protein levels in the 6KQ mutant yet are enriched in the 6KQ/6KR mass spec experiment. The explanation for this apparent contradiction is unsatisfying and fuzzy. The authors should be more precise about their data interpretation/explanation regarding this issue".

Upon reflection we agree with the reviewer that we did not discuss these interesting results in enough detail. The connection between chaperone-client interaction and client interaction is not linear. There are many clear cases where prevention of chaperone-client interaction triggers client infolding and degradation (Sluder et al. 2018). Chaperones can also target proteins for degradation. For example, phosphorylation of Ssa1 on Threonine 36 promotes Ssa1-Cln3 interaction and Cln3 degradation (Truman et al. 2012). We have revised the text to include discussion of this point.

"Western blots bands were not quantified, and there is no mention of reproducibility. In several instances, inferences are made about the different protein levels and what it might mean. Most remarkably in figure 5C HA-Rsp0A protein levels appear decreased in 6KQ, but so does the control protein. The authors should provide quantification and ratioing of band intensities to support their statements about the lower steady state levels of Rps0 in Ydj1-6KQ cells or avoid making quantified statements about qualitative observations".

We agree with the reviewer and have now quantified the Western Blots where appropriate.

"*Care should be taken when writing about the conclusion that acetylation is a direct and definitive cause of observed phenotypes. Because acetylation is inferred, but not directly measured, authors should attribute observations to mutants, acetylated / unacetylated mimetics, etc. See Lines 174 and 277*".

We agree with the reviewer and have altered the text to be more careful when addressing acetylation.

"*The authors should consider re-ordering the presentation such that Ssa1 related results are presented together, rather than jumping from Ssa1 to Ribosomes and then back to Ssa1*".

When preparing the manuscript, we underwent the same thought process the reviewer had regarding the ordering of the results. Ultimately we decided to settle for a gradual transition from cellular results (phenotypes, proteomics etc) to in vitro properties of Yd1.

"The authors should explain why they used two Gene Ontology search methods and the purpose of each".

We agree the GO was a bit confusing and have revised the figures and text in this section.

"There are many proofreading errors, as noted below, references to the incorrect figure/table, and ill-defined acronyms (e.g., BLI, GAPDH, NBD etc.) that made the manuscript challenging to read and review".

We have revised the manuscript to address these typographical issues.

Line 37-38: It is too speculative that Ydj1 acetylation affects transfer of Ydj1 from Ssa1-to Hsp82

Our revised data including the new Ydj1-Hsc82 IP clearly show that KQ Ydj1 has a decreased preference for Ssa1 and an enhanced preference for binding Hsc82. We have revised the manuscript, but feel this speculation is a valid point.

Line 106-107: Sentence does not end properly; add "previous publications"
Line 122: Why specifically mention K24Q but none of the other K##X mutations? Consider revising sentence to read "…K23Q, K24Q and other lysine mutants…"
Line 173 vs. Line 311: 63% vs 68% unaffected. Why are the values inconsistent?
Line 188: missing reference
Line 190: define NBD

Line 202: confirm accuracy of reference #32
Line 209: confirm accuracy of figure number (Figure 4A)
Line 253: possible typo, check mutant names
Line 265: typo 6KQ
Line 269: extra ")"
Line 274: define BLI
Line 297: label HPD in Figure 1
Line 304 and Line 388: mutations K23Q and K37Q are not equivalent for Ssa1 binding
Line 322: explain what is meant by "basally" acetylated
Line 323-332: as an improvement to the current model, it would be useful to include speculation about whether Ssa1 and Hsc82 are competing for the same interface that is subject to acetylation or whether acetylation is regulating complex assembly by another mechanism.
Line 357: Consider starting new paragraph
Line 445: Sentence structure and typos make sentence confusing ("primary antibody" twice).
Line 445: "(Supplementary Data 5)" is the wrong table name
Line 475: Please provide more detail about this method. Was Ssa1 washed away?
Line 552: Data Availability. Files do not yet appear to have been deposited in MassIVE or PRIDE.
Lines 574: author contributions to figures 2-5 contradict the author contributions to figure 4 (line 575)
Line 722: Ydj1-dimer ??
Line 743 (and throughout text): Please use nomenclature that more accurately defines the C-terminal tagged protein (e.g., $FLAG_C$-YDJ1 or YDJ1-FLAG).
Line 763: extra "was"
Line 783: define GAPDH
Lines 795-796: Legend does not agree with figure. There is no figure 6D
Figure 1: include information about the location of HPD on the schematic and/or structure.
Figure 4B: Rps0A is missing. Increase font size if possible.
Figure 5E: Include y-axis units

We apologize for these mistakes and have corrected these in the revised manuscript.

Response to Reviewer 4 comments

"The authors hold the conservation of the lysine sites as evidence of their functional importance but there is striking sequence conservation of the entire J domain (the amino acid sequence of the entire lysine-containing stretch is nearly perfectly conserved. This is worth pointing out to temper the notion that conservation is evidence of importance of the modifications themselves".

The entire J-domain is highly conserved in eukaryotes due to its importance in the Hsp70-J-protein interaction. It is thus likely that PTMs on this region that impact charge will alter Ydj1 function. Our previous analysis of PTMs on Hsp70 demonstrates that important PTMs are conserved throughout nature (Nitika et al. 2022; Truman et al. 2012).

"Two of the acetyl-mimicking substitutions, K23Q and K37Q, show defects under heat stress and K37Q to caffeine exposure, but neither showed any sensitivity to SDS, calcofluor white, UV, or what I think is hydroxyurea (HU?) treatment. What 'HU' stands for or why it is relevant is not mentioned anywhere in the paper (text, legends, methods, etc)".

The author raises a valid point and we apologize for not explaining this in a clearer fashion. HU, hydroxyurea is a DNA replication inhibitor in yeast and mammalian cells. Our previous studies determined that Ydj1 (and its mammalian counterpart DNAJA1) bind and stabilize subunits of the ribonucleotide reductase complex (RNR) (Sluder et al. 2018). Loss of Ydj1 function produces hydroxyurea sensitivity and thus was examined in this study. We have revised the text to reflect this.

“That wildtype Ydj1 expression did not affect growth under the heat stress and caffeine conditions suggests that this acetylation is not occurring at all or at least is not happening at sufficient levels to prevent Ydj1 from acting under these conditions. If this is the case, then it would contradict the conclusions of the paper. The authors include this in the discussion “Consistent with our original hypothesis, acetylation of Ydj1 indeed disrupted interaction with Ssa1”.

Our cellular and in vitro data indicate that acetylation does disrupt Ssa1-Ydj1 interaction. In this sense we absolutely agree with the reviewer that in WT cells there must be either a low stoichiometry of acetylation or that acetylation is very tightly partitioned.

“Additionally, it is not clear how many times these experiments were done, and no quantification of the growth was undertaken. In Figure 3A, the abundance of each Ydj1 protein is shown but it is also not clear how many times this was done (no N value in the methods or legend) and no quantification was undertaken”.

We have now revised this in the text and figures.

“It does not appear that the authors attempted to measure acetylation of the Ydj1 protein in the yeast lysates in the first place. It is all based on proteomic reports which seems insufficient in light of the confusing data presented in Figure 2 and 3. If acetylation is happening on K23 and K37, then it should be detectable even by western blot with IPed material”.

We selected the six residues to study based on their previous detection in proteomic studies. As explained in the text of the manuscript this can be viewed here: https://intrinsicdisorder.com/a/ptm_png.py?I=YNL064C. Each of the six acetylation sites have been detected between 17 and 42 times. An important point to note is that throughout our twenty-year study of the chaperone code we see clear evidence that sites that impact specific stress resistance are intriguingly often not those that are activated by the same stresses. This is possibly due to multi-pathway crosstalk and integration. However, our typical methodology involves identifying interesting sites and then studying 1) their impact by mutation and 2) their regulation using PTM-specific antibodies. We initially attempted to analyze the six sites using general acetyl-lysine antibodies, but the data were confounded by the poor quality of the antibody and multiple acetylated sites. We then attempted to generate site specific acetyl-lysine antibodies twice for Ydj1 at great cost and time ($5000 for each one, nine-month generation time). Disappointingly none of these antibodies were selective enough for us to use, they simply detected total Ydj1. As such, we ended up with the analysis we present here. We believe that the identification of these sites by MS multiple times (in both yeast and human cells) is sufficient for this study.

“A final source of confusion with figure 2 is that the K6Q mutant fails to restore growth under any of the stress conditions”.

The 6KQ mutant is the most defective for chaperone activity, probably due to its inability to bind Ssa1.

“*In apparent contradiction of the conclusions that will ultimately be reached later, there is only a minor difference in the recovery of Ssa1 and Ssa2. In fact, the change is below the threshold of -1 or 1 identified by the authors. If the K37Q substitution (contained in the even more severely impaired K6 mutant) ablates the interaction with Ssa1 (as is shown in Figure 6), why is there such a small difference in the interaction in the proteomic experiment?”*

We bring up this exact point in the manuscript. Ssa1 and Ssa2 have such a high degree of identity that they are very challenging to tell them apart at the proteomic level. To bypass this issue we carried out a Ydj1-Ssa1 IP probing for an antibody with specificity to Ssa1. This IP demonstrated a clear acetylation-

mediated Ssa1-Ydj1 interaction. Our *in vitro* experiments confirm the results of our structural prediction and IP.

"The authors measure translational fidelity in strains expressing Ydj1 mutants but these results are not compared to what seem like obvious controls like deletion strains of Gcd11, Rps3, and Rps0. I think this should be done to contextualize the magnitude of the difference in GUU utilization".

These proteins are essential for cell viability and so the above experiment is unfortunately not possible.

"Figure 6 goes on to measure very specific properties of the Ydj1 mutants (but not all of them) – refolding, ATPase activity with Ssa1, and interaction with Ssa1. The results are somewhat consistent but there are key differences that undermine the conclusions and are not really sufficiently discussed. In the model based on PDBID: 5NRO, were the surfaces on Hsp70 that are near K37 examined?".

We have generated revised models using AlphaFold 3 and have revised Fig. 6 containing the in vitro data.

"Is there some other reason to think that loss of the positive charge (with the Q substitution) might result in the loss of interaction with Ssa1 (but not Ssa2)? There is a negatively charged residue in the bacterial structure but I have no idea if this is present in the yeast structure. Some more detailed comparison of what that interface might look like would be easy to do and help understand what is going on with that mutant".

While we do not have the resources to examine this experimentally, we have modeled the interface between Ydj1 and Ssa1-4. There are some interesting sequence differences in the Ssas over that region that may suggest Ydj1 acetylation may differentially impact the binding of Ssa isoforms. We have included these models as supplemental figure S2.

"Despite this, there is the statement "Altogether these in vitro results confirm the in vivo results demonstrating that acetylation of K37 renders Ydj1 inactive and further show that acetylation inhibits the cochaperone activity of Ydj1 because it is unable to interact with Ssa1." How then is the K37Q mutant able to function under CFW, SDS, HU, and UV stress?"

The reviewer brings up an excellent point. The answer to this question may lie in the reviewer's above comment. The in vitro Ydj1 experiments were carried out with only Ssa1, but in the yeast mutants ALL Ssa isoforms are present. Ydj1 acetylation may act in an Ssa-isoform-dependent manner. It is possible that while K37 acetylation (and K37Q) prevent Ydj1-Ssa1 interaction, the same may not be true for Ydj1-Ssa2/3/4. We have added this idea to the discussion and the models are present now as Fig S2B and S2C.

"*Figure 1. Include information on how the Alphafold2 structure was made (The authors state that the structure shown in Figure 1B was generated using Alphafold2 but there is no information provided on how this was done. Did the authors use a custom workflow? The structure shown does not resemble the one in the Alphafold database. All the details regarding the generation of this structure should be provided in the methods and the structure file should be provided as a supplementary file.)*"

We agree with the reviewer that this was not clear in our original submission. With the release of AlphaFold 3 we have remodeled the structures and have added these to the manuscript.

"Figure 2. Replicates and quantitation of growth".

We have revised this section

“Figure 3. Either much more explanation, or much less attention to this”.

We have revised this figure

“*Figure 4. The figures are very low resolution and I had to look at the supplementary excel sheet to see what was happening*”.

We apologize for this issue, this is probably and artifact of the journal submission process. We have revised this figure and hope it is clearer for the reviewers.

Figure 4D should be complemented with more detailed information and analysis of the interaction model – particularly as it pertains to K37. Some discussion of how Ssa1, 2, 3, and 4 differ in this area should also be included. Figure 4E should include an analysis of Ssa2 (and 3 and 4 for that matter).

While we agree exploring the impacts of these sites on Ssa1, 2, 3 and 4 interaction would be fascinating, these go beyond the scope of our original project. We simply do not have the resources to do these experiments at this current time. However, we have revised the discussion section and have mentioned these ideas.

“*Figure 5. Should include experiments with yeast lacking the three ribosomal genes as controls*”.

The ribosomal proteins identified in figure 5 are essential for cell viability and thus we could not create the suggested deletion strains. However, We believe that cells aching Ydj1 function serve as a reasonable control for determining the impact of acetylation site mutants on translation.

We hope that these responses and the revised manuscript are satisfactory. Again, we thank the Editor and Reviewers for their careful reading and valuable feedback. We feel this process has led to a vastly improved manuscript.

Sincerely,

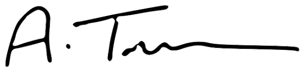

Andrew Truman, PhD
Professor, Biological Sciences, University of North Carolina at Charlotte

References

Caplan, A. J., J. Tsai, P. J. Casey, and M. G. Douglas. 1992. “Farnesylation of YDJ1p Is Required for Function at Elevated Growth Temperatures in Saccharomyces Cerevisiae.” *The Journal of Biological Chemistry* 267 (26): 18890–95.

Flom, Gary A., Marta Lemieszek, Elizabeth A. Fortunato, and Jill L. Johnson. 2008. “Farnesylation of Ydj1 Is Required for in Vivo Interaction with Hsp90 Client Proteins.” *Molecular Biology of the Cell* 19 (12): 5249–58.

Sluder, Isaac T., Nitika, Laura E. Knighton, and Andrew W. Truman. 2018. “The Hsp70 Co-Chaperone Ydj1/HDJ2 Regulates Ribonucleotide Reductase Activity.” *PLoS Genetics* 14 (11): e1007462.

Sojourner, Samantha J., Willie M. Graham, Aurellia M. Whitmore, Jana S. Miles, Devon Freeny, and Hernan Flores-Rozas. 2018. “The Role of HSP40 Conserved Motifs in the Response to Cytotoxic Stress.” *Journal of Nature and Science* 4 (4). https://www.ncbi.nlm.nih.gov/pubmed/29682607.

Truman, Andrew W., Kolbrun Kristjansdottir, Donald Wolfgeher, Naushaba Hasin, Sigrun Polier, Hong Zhang, Sarah Perrett, Chrisostomos Prodromou, Gary W. Jones, and Stephen J. Kron. 2012. “CDK-Dependent Hsp70 Phosphorylation Controls G1 Cyclin Abundance and Cell-Cycle Progression.” *Cell* 151 (6): 1308–18.
