# Supplementary material for: Acetylation of the yeast Hsp40 chaperone protein Ydj1 fine-tunes proteostasis and translational fidelity
Source: PLoS Genet. 2024 Dec 9;20(12):e1011338. doi: 10.1371/journal.pgen.1011338 (PMC11658694; doi:10.1371/journal.pgen.1011338)
Supplement: Attachment — Submitted filename: Review of Ydj1 aceylation paper_final.docx [file pgen.1011338.s005.docx]

Review of “Acetylation of the yeast Hsp40 chaperone protein YDJ1 fine-tunes proteostasis and translational fidelity”

Summary.

This paper utilizes the model organism yeast *Saccharomyces cerevisiae* to investigate the potential role of acetylation within the J-domain of HSP40 protein Ydj1. The authors use a wide range of *in vivo* and *in vitro* assays to evaluate whether substitution of conserved acetylated lysine residues with arginine (unacetylated mimetic) or glutamine (acetylation mimetic) changes Ydj1 associated phenotypes including growth under stress conditions, binding and activating the Hsp70 Ssa1, and protein translation. The research takes advantage of multiple approaches to address a complex problem: how do post-translational modifications regulate a protein with diverse important cellular functions? The strongest data presented comes from the work with Ydj1-Ssa1 interactions, while the effects of acetylation mimetics on protein translation are not as well presented. Overall, the paper requires edits to address issues with data interpretation and significant proof reading before it can be considered for publication.

Major criticisms:

1) In Figure 3 the argument is made that doublet protein bands visible by Western blot can be attributed to farnesylation. However, the C-terminal FLAG tag would cause interference with farnesylation. This does not change the main conclusions of the paper, but any comments about Ydj1 doublets with respect to farnesylation need to be significantly revised or removed. See Lines 140 and 366.
2) Figure 5A and 5C and its interpretation: The authors observe that 3 proteins (Rsp0A, Rps3 and Gcd11) have lower protein levels in the 6KQ mutant yet are enriched in the 6KQ/6KR mass spec experiment. The explanation for this apparent contradiction is unsatisfying and fuzzy. The authors should be more precise about their data interpretation/explanation regarding this issue.
3) Western blots bands were not quantified, and there is no mention of reproducibility. In several instances, inferences are made about the different protein levels and what it might mean. Most remarkably in figure 5C HA-Rsp0A protein levels appear decreased in 6KQ, but so does the control protein. The authors should provide quantification and ratioing of band intensities to support their statements about the lower steady state levels of Rps0 in Ydj1-6KQ cells or avoid making quantified statements about qualitative observations.
4) Care should be taken when writing about the conclusion that acetylation is a direct and definitive cause of observed phenotypes. Because acetylation is inferred, but not directly measured, authors should attribute observations to mutants, acetylated / unacetylated mimetics, etc. See Lines 174 and 277.
5) The authors should consider re-ordering the presentation such that Ssa1 related results are presented together, rather than jumping from Ssa1 to Ribosomes and then back to Ssa1.
6) The authors should explain why they used two Gene Ontology search methods and the purpose of each. Both methods should be expanded upon in the methods. The resulting data from these analyses (i.e., Figure 4C) is challenging to understand and needs further explanation in the narrative or legend (e.g., What are the dotted circles and what does it mean if a protein is outside the circle?)
7) There are many proof reading errors, as noted below, references to the incorrect figure/table, and ill-defined acronyms (e.g., BLI, GAPDH, NBD etc.) that made the manuscript challenging to read and review.

Minor criticisms:

Line 37-38: It is too speculative that Ydj1 acetylation affects transfer of Ydj1 from Ssa1-to Hsp82

Line 106-107: Sentence does not end properly; add “previous publications”

Line 122: Why specifically mention K24Q but none of the other K##X mutations? Consider revising sentence to read “…K23Q, K24Q and other lysine mutants…”

Line 173 vs. Line 311: 63% vs 68% unaffected. Why are the values inconsistent?

Line 188: missing reference

Line 190: define NBD

Line 202: confirm accuracy of reference #32

Line 209: confirm accuracy of figure number (Figure 4A)

Line 253: possible typo, check mutant names

Line 265: typo 6KQ

Line 269: extra “)”

Line 274: define BLI

Line 297: label HPD in Figure 1

Line 304 and Line 388: mutations K23Q and K37Q are not equivalent for Ssa1 binding

Line 322: explain what is meant by “basally” acetylated

Line 323-332: as an improvement to the current model, it would be useful to include speculation about whether Ssa1 and Hsc82 are competing for the same interface that is subject to acetylation or whether acetylation is regulating complex assembly by another mechanism.

Line 357: Consider starting new paragraph

Line 445: Sentence structure and typos make sentence confusing (“primary antibody” twice).

Line 445: “(Supplementary Data 5)” is the wrong table name

Line 475: Please provide more detail about this method. Was Ssa1 washed away?

Line 552: Data Availability. Files do not yet appear to have been deposited in MassIVE or PRIDE.

Lines 574: author contributions to figures 2-5 contradict the author contributions to figure 4 (line 575)

Line 722: Ydj1-dimer ??

Line 743 (and throughout text): Please use nomenclature that more accurately defines the C-terminal tagged protein (e.g., FLAG_C_-YDJ1 or YDJ1-FLAG).

Line 763: extra “was”

Line 783: define GAPDH

Lines 795-796: Legend does not agree with figure. There is no figure 6D

Figure 1: include information about the location of HPD on the schematic and/or structure.

Figure 4B: Rps0A is missing. Increase font size if possible.

Figure 5E: Include y-axis units
